# Supplementary material for: NQO1 targeting prodrug triggers innate sensing to overcome checkpoint blockade resistance
Source: Nat Commun. 2019 Jul 19;10:3251. doi: 10.1038/s41467-019-11238-1 (PMC6642086; doi:10.1038/s41467-019-11238-1)
Supplement: Supplementary file 1 — Supplementary Information [file 41467_2019_11238_MOESM1_ESM.pdf]

Supplementary Fig. 1

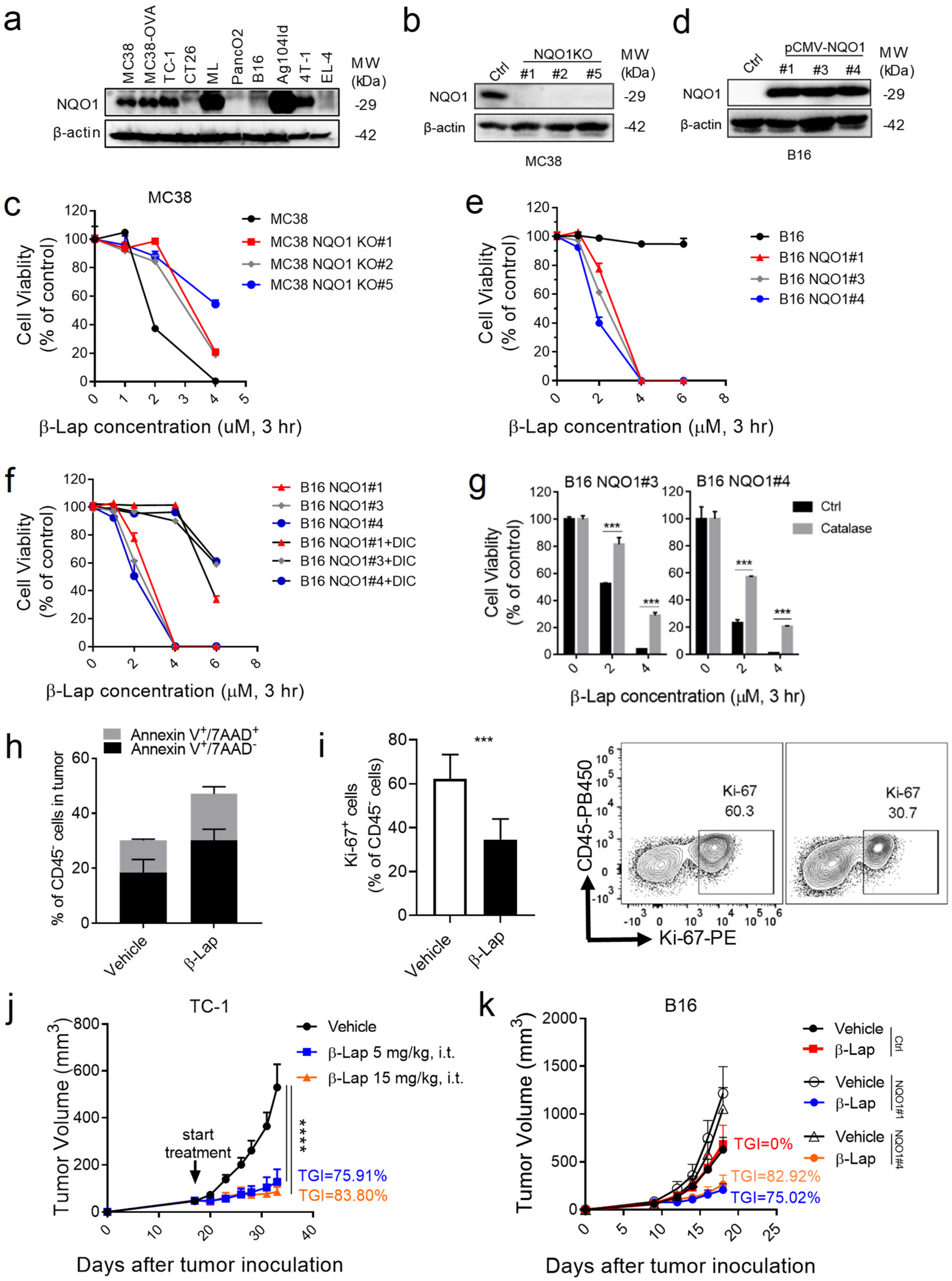

Supplementary Fig. 1 β-Lap kills murine tumor cells in an NQO1-dependent manner. (**a**) NQO1 expression in different murine cancer cell lines was determined by western blotting assay. (**b)** NQO1 expression in different clones of MC38 cells with CRISPR-based NQO1 Knockout was determined by western blotting assay. (**c**) MC38 cells (NQO1 WT or KO) were treated with β-lap for 3 hr followed by washing and replacing fresh medium. Cell viability was determined by Sulforhodamine B (SRB) Assay 48 hr later. (**d**) NQO1 expression in different clones of B16 cells stably harboring a pCMV-NQO1 expression vector was determined by western blotting assay. (**e, f**) B16 cells (NQO1 null or stable overexpression) were treated with β-lap with or without dicoumarol (DIC, 50 μM) for 3 hr followed by washing and replacing medium. Cell viability was determined by SRB Assay 48 hr later. (**g**) NQO1 overexpressing B16 cells (clone #3 and #4) were exposed to β-lap for 3 hr. Catalase (1000 U/ml) was added and cell viability was assessed 48 hr later. (**h, i**) C57BL/6 mice bearing MC38 tumor (n=6 for vehicle group, n=8 for β-lap group) were locally treated with β-lap (15 mg/kg, i.t.). 18 hours after treatment, tumor tissues were processed into single cell suspension and CD45- cells were stained with 7AAD/Annexin V in **h** or Ki-67 in **i** followed by flow cytometry analysis. (**j**) C57BL/6 mice (n=5/group) were transplanted with TC-1 cells, and intratumorally treated with β-lap (5 or 15 mg/kg) every other day for four times. Tumor growth was monitored twice a week. **(k**) C57BL/6 mice were transplanted with parental B16 cells (NQO1 null, n=4/group) or NQO1 stable overexpressing B16 cells (clone #1, n=4/group and clone #4, n=5/group) and treated with β-lap (15 mg/kg, i.t.) every other day for four times. Data are shown as mean ± SEM. **p < 0.01, ***p < 0.001, ****p < 0.0001 determined by unpaired student t-test in **g** or two-way ANOVA in **h** and **i**.

Supplementary Fig. 2

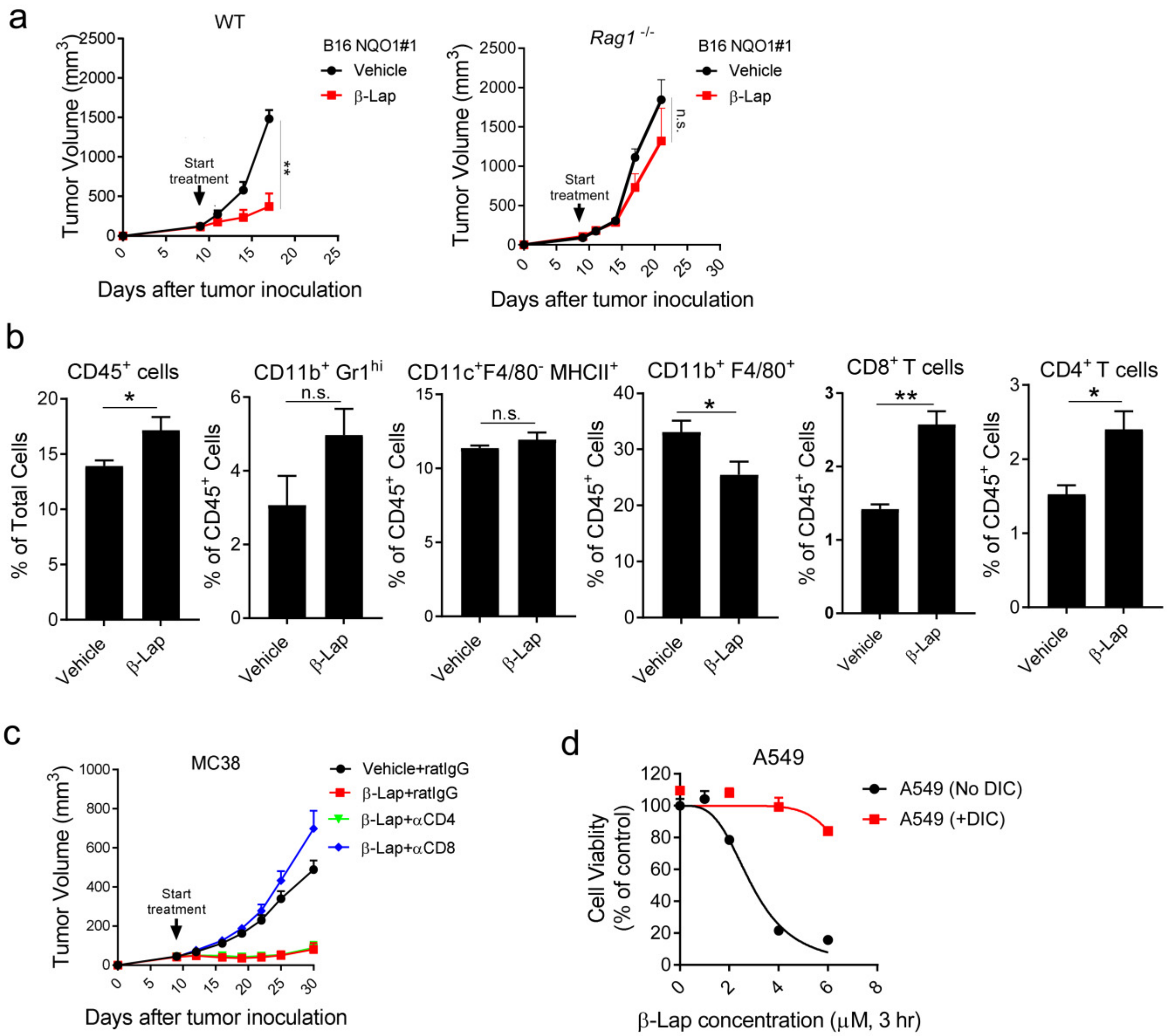

Supplementary Fig. 2 β-Lap-mediated antitumor effect depends on T cells. (**a**) NQO1 overexpressing B16 cells (B16 NQO1 #1) were subcutaneously transplanted into C57BL/6 WT and *Rag1*$^{-/-}$ mice (n=5/group), respectively. Tumor bearing mice were treated with β-lap (15 mg/kg, i.t.) every other day for four times. (**b**) Changes of the immune cells in the tumor microenvironment 7 days after β-lap treatment. C57BL/6 mice (n=4/group) were transplanted with MC38 cells and intratumorally treated with 15 mg/kg of β-lap for twice, and 7 days after the last treatment, tumor tissue was removed and digested, and immune cells were analyzed with flow cytometer. (**c**) C57BL/6 mice (n=5/group) were transplanted with MC38 cells and treated with β-lap (15 mg/kg, i.t.) every other day for four times. For T cell depletion, 200 µg of anti-CD4 or anti-CD8 antibodies were injected four times at three days interval during the treatment. (**d**) NQO1 positive human lung carcinoma lines A549 grown in 48-well plates were exposed to β-lap (0-6 µM) ± dicoumarol (DIC, 50 µM) for 3 hr and cell survival was assessed 4 days later with SRB Assay. Data are shown as mean ± SEM. (unpaired student t-test was used to analyze the significance of changes, $^{*}p < 0.05$, $^{**}p < 0.01$).

Supplementary Fig. 3

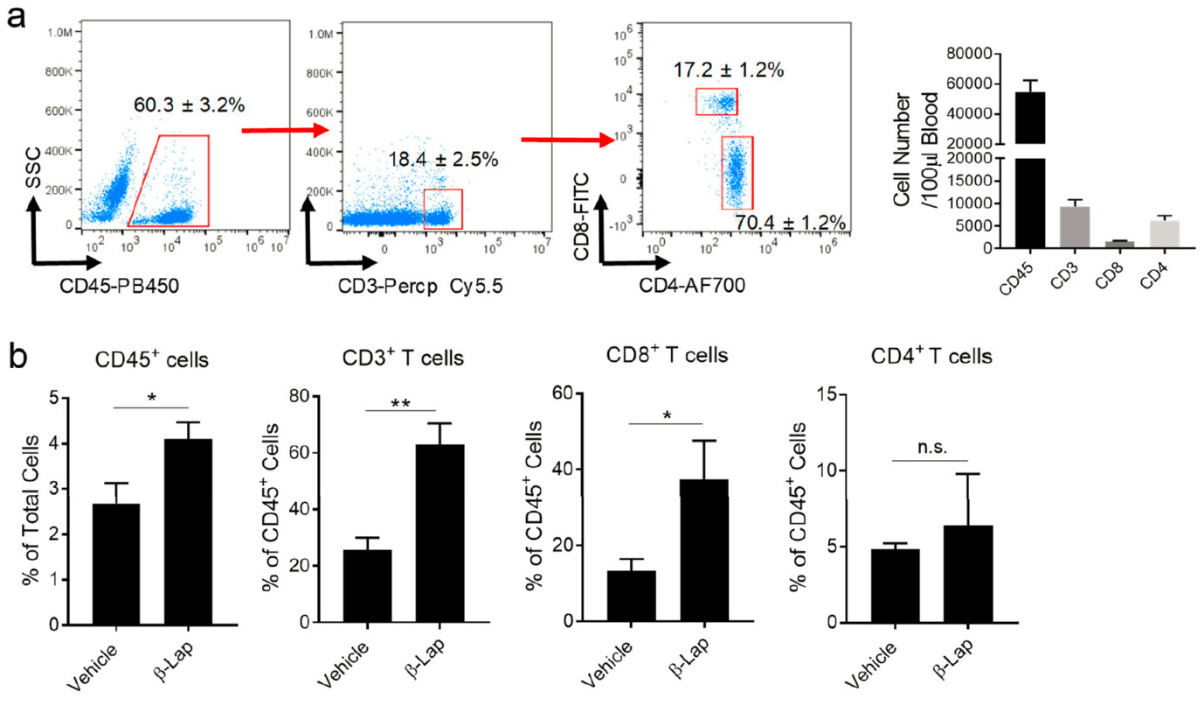

Supplementary Fig. 3 NSG-SMG3 humanized mouse model. (**a**) 4-week-old NSG-SGM3 mice (n=12) were irradiated 1 Gy and injected 1x10^5 human $CD34^+$ cells by tail vein. 11 weeks after reconstitution, peripheral blood were collected and human $CD45^+$, $CD3^+$, $CD8^+$ and $CD4^+$ T cells were determined by flow cytometry assay. Representative flow gating strategy (up) and quantification of cell numbers were shown (down). (**b**) The immune cells changes in the tumor microenvironment after β-lap treatment. NSG-SMG3 humanized mice (n=5 for vehicle group, n=6 for β-lap treatment group) were s.c. transplanted with A549 cells. Tumor bearing mice (about 60 mm^3) were intratumorally treated with 10 mg/kg of β-lap for four doses, and 12 days after the last treatment, tumor tissue was removed and digested, and human $CD45^+$, $CD3^+$, $CD8^+$, and $CD4^+$ immune cells were analyzed with flow cytometer. Data shown as mean ± SEM. Unpaired student t-test was used to analyze the significance of changes, *$p < 0.05$; **$p < 0.01$.

Supplementary Fig. 4

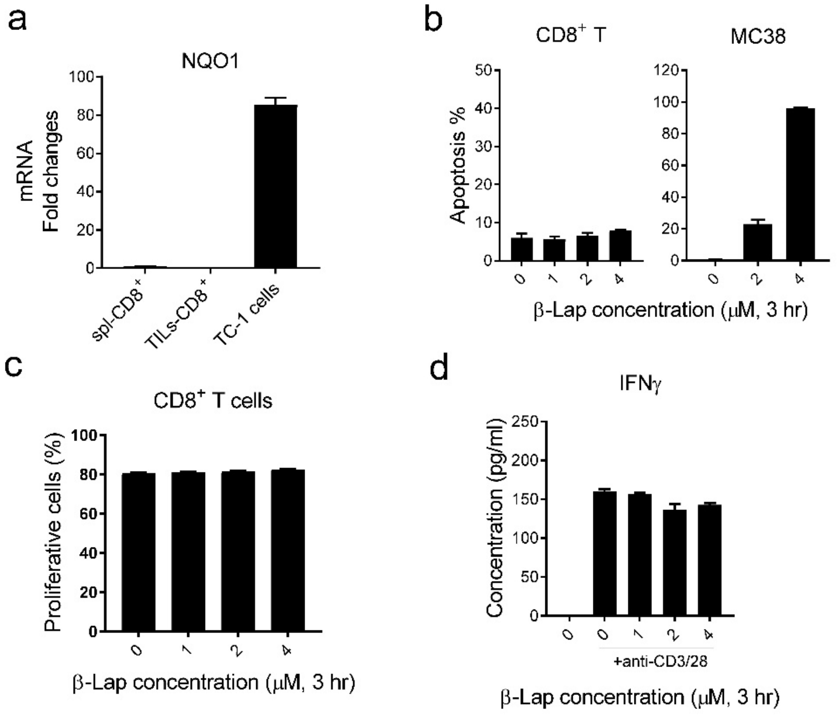

Supplementary Fig. 4 β-Lap has no effect on CD8 T cell survival and activation. (**a**) Naïve $CD8^+$ T cells and tumor-infiltrating CD8 T cells were separately sorted out from the spleen of naïve C57BL/6 mice or from the tumor tissue of TC-1 tumor-bearing mice (n=3/group). The expression level of NQO1 in $CD8^+$ T cells was determined by quantitative Real-Time PCR. NQO1-positive TC-1 cell was used as a control. (**b**) The splenocytes from naïve mice (n=4/group) were exposed to different concentration of β-lap (4 μM) for 3 hr followed by washing and replacing medium. 24 hr later, cells were stained with 7-AAD and Annexin V and related immune cell markers followed by flow cytometry analysis. MC38 cells (n=3/group) with the same treatment was used as a control. (**c**) CFSE labeled splenocytes (n=4/group) were treated with different concentration of β-lap for 3 hr followed by washing and stimulation with anti-CD3 (1 μg/ml) and anti-CD28 (2μg/ml) for 48 hr. The proliferative CD8 T cells were determined by flow cytometry assay. (**d**) Splenocytes (n=3/group) were exposed to β-lap for 3 hr followed by washing and stimulation with anti-CD3 (1 μg/ml) and anti-CD28 (2 μg/ml) for 48 hr. IFNγ production was analyzed by Cytometric Bead Array (CBA) mouse IFNγ assay. The data are shown as mean ± SEM from three independent experiments.

Supplementary Fig. 5

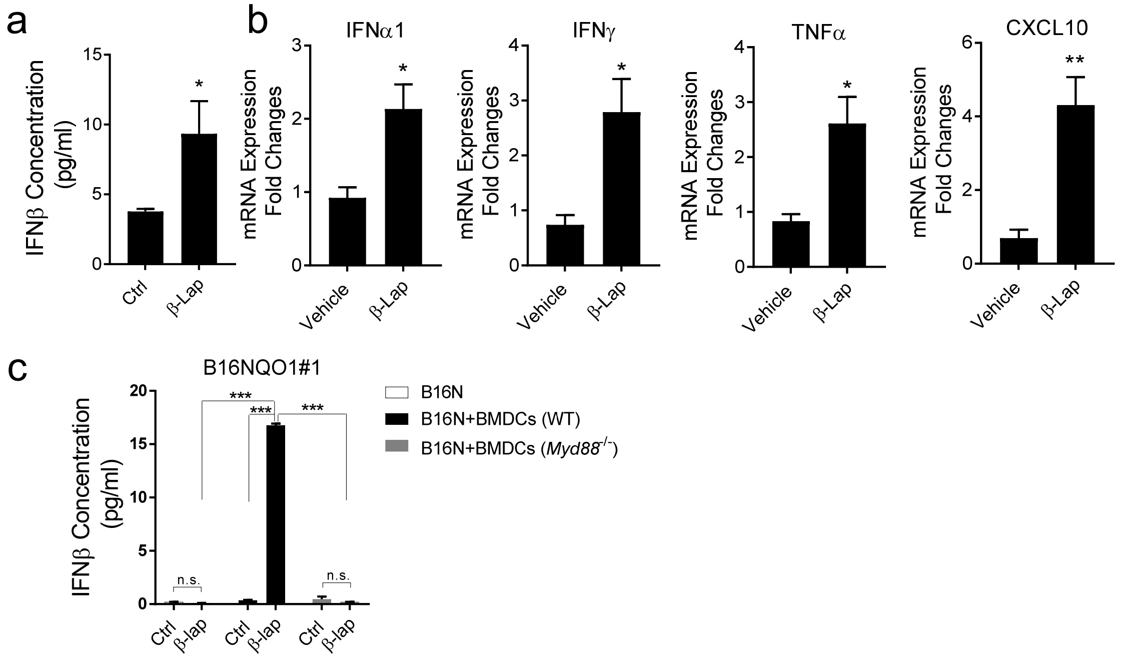

Supplementary Fig. 5 β-Lap treatment induces type I IFN production. (a, b) MC38-OVA bearing mice (n=3/group) were treated with β-lap (15 mg/kg, i.t.) for two times. Six days later, tumor tissues were collected for both single cell digestion and RNA extraction. The secreted IFNβ from the suspended cell supernatant was measured by ELISA after a 24 hr culture in **a**. The mRNA levels of IFNα1, IFNγ, TNFα and CXCL10 were determined by real-time PCR assay in **b**. (**c**) NQO1-overexpressing B16 cells (B16NQO1#1) were treated with β-lap (4 μM) for 3 hr followed by washing and replacing fresh medium. 24 hr later, BMDCs from WT or *Myd88*$^{-/-}$ mice were cocultured with β-lap-treated tumor cells for another 48 hr. The level of IFNβ from the culture supernatant was detected by ELISA. Data are shown as mean ± SEM from two independent experiments. *p < 0.05, **p < 0.01, ***p < 0.001 determined by unpaired t-test.

Supplementary Fig. 6

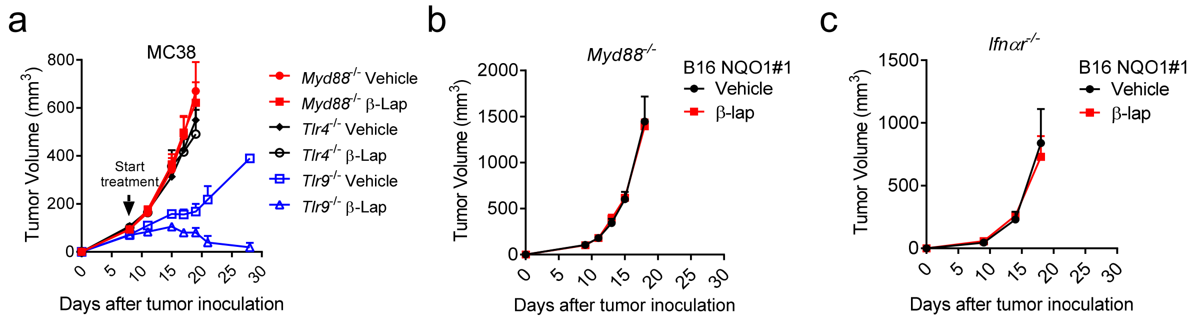

Supplementary Fig. 6 TLR4/MyD88/type I IFNs pathway is required for the antitumor effect of β-lap. (**a**) MC38 cells were implanted into *Myd88*$^{-/-}$ (n=6/group), *Tlr4*$^{-/-}$(n=4/group) and *Tlr9*$^{-/-}$ (n=3/group) C57BL/6 mice, respectively. Tumor bearing mice were then treated with β-lap (15 mg/kg, i.t.) every other day for four times. (**b, c**) NQO1-overexpressing B16 cells were inoculated in *Myd88*$^{-/-}$ (b, n=3 for vehicle group, n=4 for β-lap group) or *Ifnar1*$^{-/-}$ mice (c, n=4 for vehicle group, n=5 for β-lap group). Tumor bearing mice were treated with β-lap (15 mg/kg, i.t.) every other day for four times. Tumor growth was monitored twice a week. Data are shown as mean ± SEM.

Supplementary Fig. 7

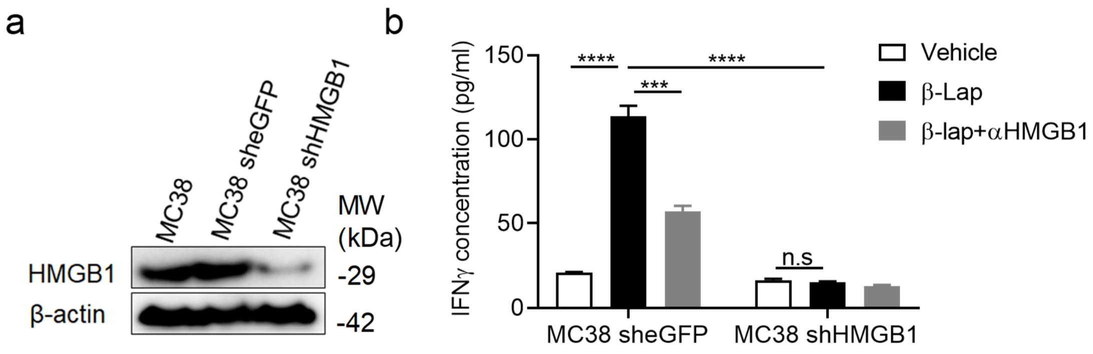

Supplementary Fig. 7 β-Lap treatment enhance T cell cross-priming in a HMGB1 dependent manner. (**a**) HMGB1 expression in different MC38 cell lines (WT MC38, MC38 sheGFP and MC38 shHMGB1 cell pool) was determined by western blotting assay. (**b**) MC38 sheGFP (n=3) or MC38 shHMGB1 cells (n=3) were treated with β-lap (4 μM) for 3 hr followed by replacing fresh medium for another 24 hr. BMDCs were exposed to 40 μg/ml OVA protein for 4 hr, then naïve OT1 $CD8^+$ T cells and the supernatants from β-lap-treated tumor cells (with/without anti-HMGB1 antibody) were added and allowed to incubate for another 48 hr. IFN-γ was determined by cytometric bead array assay. Data shown as mean ± SEM. Unpaired student t-test was used to analyze the significance of changes, ***$p < 0.001$, ****$p < 0.0001$.

Supplementary Fig. 8

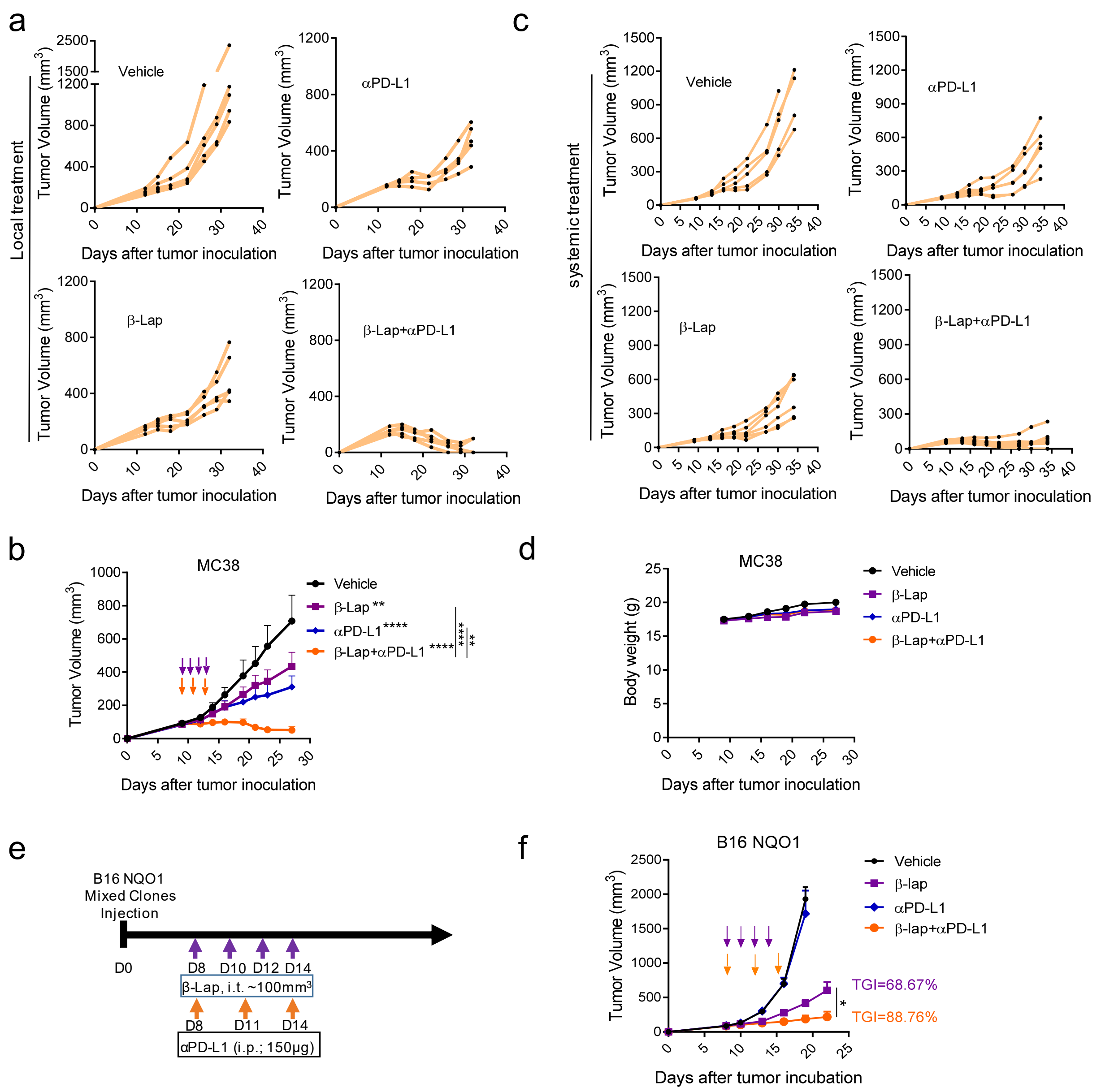

Supplementary Fig. 8 β-lap treatment can synergize with anti-PD-L1 immune checkpoint blockage. (a) MC38 tumor cells were s.c. inoculated into the flank of C57BL/6 mice (n=5/group). Mice bearing advanced tumor (about 150-200 mm3) were locally treated with β-lap (15 mg/kg, i.t.) for four times with or without anti-PD-L1 based checkpoint blockade (100 µg, i.p.) for three times. Tumor growth was monitored twice a week, and the growth curve of the individual mouse in each group was shown. (b) MC38 tumor cells were s.c.inoculated into the flank of C57BL/6 mice (n=5/group). Mice bearing advanced tumor (about 100 mm3) were locally treated with low dose of β-lap (5 mg/kg, i.t.) for four times with or without PD-L1 based checkpoint blockage. (c, d) MC38 tumor bearing mice (about 50-100 mm3) were systematically treated with β-lap (30

mg/kg, i.p.) for six times with or without PD-L1 based checkpoint blockage (n=5/group for vehicle treatment; n=6/group for β-lap and vehicle+anti-PD-L1; n=8/group for β-lap+anti-PD-L1 treatment). Tumor growth in (c) and body weight (d) were monitored twice a week. Growth curve of the individual mouse in each group was shown. (e, f) B16 cells with stable NQO1 overexpression (Mixed clone #1, #3 and #4) were s.c. inoculated into C57BL/6 mice. Tumor bearing mice (about 100 mm3) were locally treated with β-lap (15 mg/kg, i.t.) for four times with or without anti-PD-L1 based checkpoint blockage (150 μg, i.p.) for three times (n=5/group for vehicle treatment, β-lap and vehicle+anti-PD-L1; n=7/group for β-lap+anti-PD-L1 treatment)). Treatment schema was shown (e) and the tumor growth curve was monitored (f). Data are shown as Mean ± SEM from at least two independent experiments. $^*p < 0.05$, $^{**}p < 0.01$, $^{***}p < 0.001$, $^{****}p < 0.0001$ determined by two-way ANOVA test.

Supplementary Fig. 9

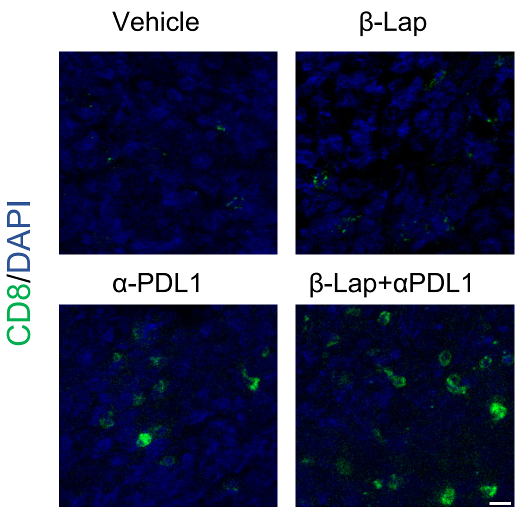

Supplementary Fig. 9 CD8 immunostaining of tumor tissues after β-lap or combined therapy with anti-PD-L1. C57BL/6 mice bearing MC38-OVA tumor were locally treated with β-lap (15 mg/kg, i.t.) every other day for four times or anti-PD-L1 (100 μg, i.p.) for three times, alone or combination. 12 days after first treatment, tumor infiltrating $CD8^+$ T cells were analyzed by immunofluorescence. Scale bar is 10 μm.

Supplementary Fig. 10

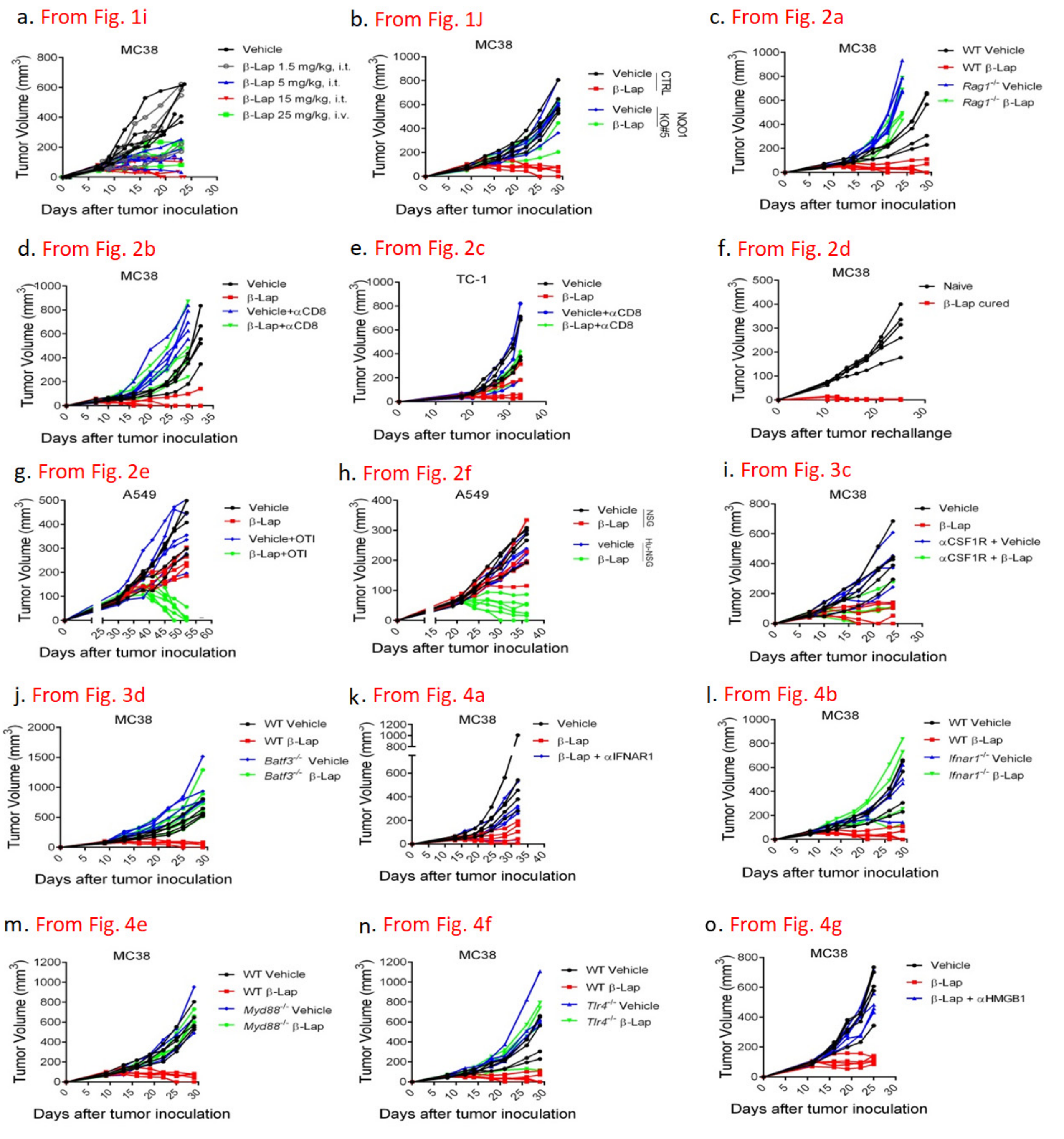

Supplementary Fig. 10 "Spaghetti plots" of each tumor growth curve of individual mouse for in vivo study. (**a, b**) The tumor growth curve of individual mouse from Fig. 1i in **a** and Fig.1 j in **b**. (**c-h**) The tumor growth curve of individual mouse from Fig. 2a in **c**, Fig. 2b in **d**, Fig. 2c in **e,** Fig. 2d in **f**, Fig. 2e, in **g**, Fig. 2f in **h**. (**i, j**) The tumor growth curve of individual mouse from

Fig. 3c in **j** and from Fig. 3d in **j**. (**k-o**) The tumor growth curve of individual mouse from Fig. 4a in **k**, Fig. 4b in **l**, Fig. 4e in **m,** Fig. 4f in **n** and from Fig. 4g in **o**.

Supplementary Fig. 11

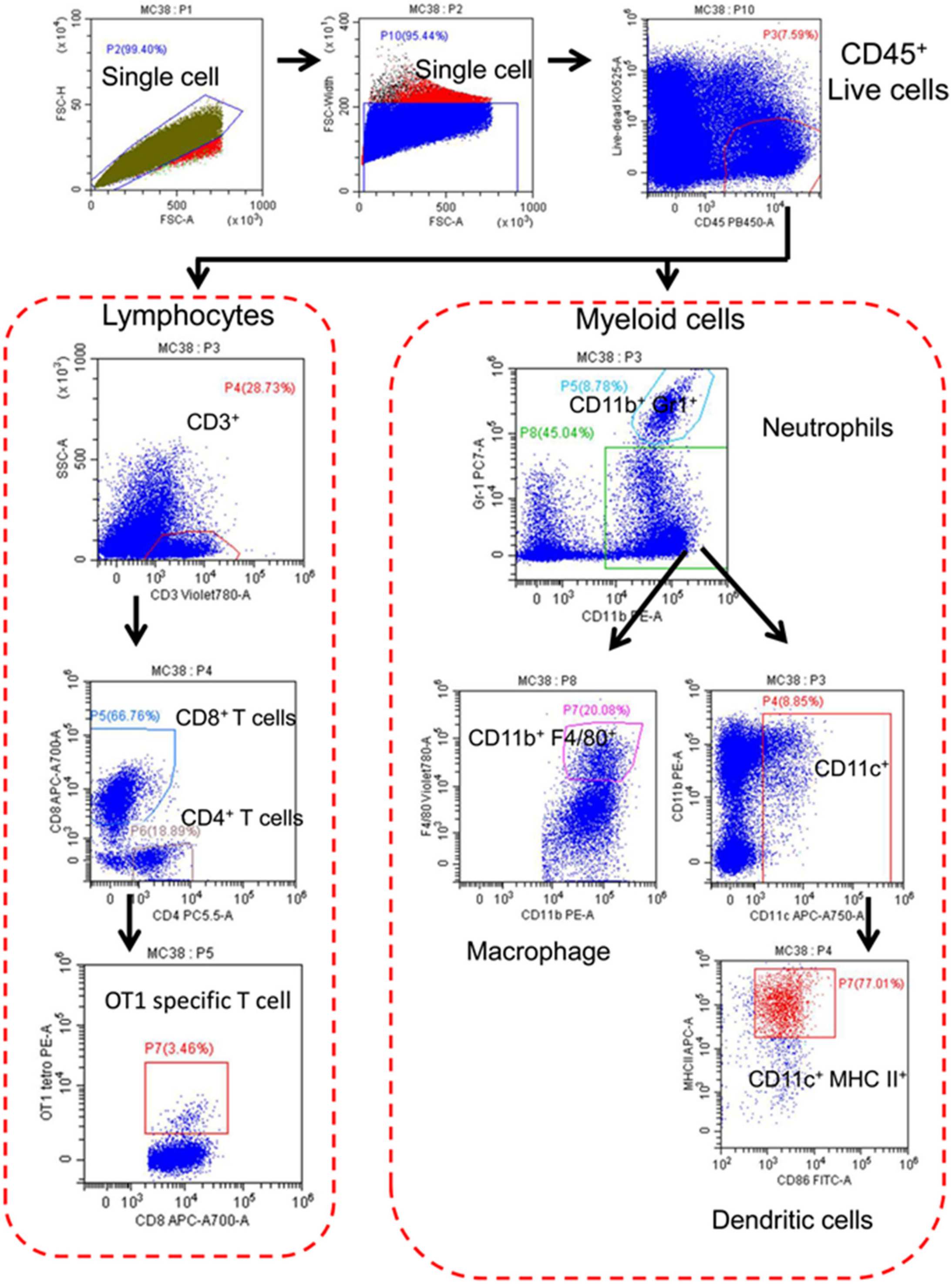

Supplementary Fig. 11 Example of gating schematics to characterize the immune cell infiltrates in tumor tissue.

Supplementary Table 1 Antibodies used in this study.

Antibodies	SOURCE	IDENTIFIER	Dilutions
InVivoMab anti-mouse CD4 (GK1.5)	BioXcell	Cat#BE0003-1	No
InVivoMab anti-mouse CD8 (YTS169.4)	BioXcell	Cat#BE0117	No
InVivoMab anti-mouse PD-L1 (10F.9G2)	BioXcell	Cat#BE0101	No
InVivoMab anti-mouse IFNAR1(MAR1-5A3)	BioXcell	Cat#BE0241	No
InVivoMab anti-mouse CSF1R(AFS98)	BioXcell	Cat#BE0213	No
InVivoMAb polyclonal rat IgG	BioXcell	Cat#BE0094	No
Anti-CD45 (FACs, 30-F11)	Biolegend	Cat#103126	1:200
Anti-CD3 (FACs, 17A2)	Biolegend	Cat#100232	1:200
Anti-CD4 (FACs, RM4-5)	Biolegend	Cat#100540	1:200
Anti-CD8a (FACs, 53-6.7)	Biolegend	Cat#100730	1:200
Anti-CD25 (FACs, PC61)	Biolegend	Cat#102008	1:200
Anti-Foxp3 (FACs, MF-14)	Biolegend	Cat#126408	1:200
iTAg Tetramer/PE - H-2 Kb OVA (SIINFEKL)	MBL	Cat# TB-5001-1	1:100
NQO1 antibody	Cell signaling	Cat# 62262	1:1000
β-actin antibody	Cell signaling	Cat# 5125	1:5000
Live/dead staining	invitrogen	Cat# 65-0866-18	1:1000

Supplementary Table 2 Primers used in this study.

Oligonucleotides
Mouse *CXCL10* forward primer: GTCCTAATTGCCCTTGGT
Mouse *CXCL10* reverse primer: TCTTGCTTCGGCAGTTAC
Mouse *IFNα1* forward primer: TCCCCTGACCCAGGAAGATGCC
Mouse *IFNα1* reverse primer: ATTGGCAGAGGAAGACAGGGCT
Mouse *IFNγ* forward primer: ACTGGCAAAAGGATGGTGAC
Mouse *IFNγ* reverse primer: ACCTGTGGGTTGTTGACCTC
Mouse *TNFα* forward primer: ACGGCATGGATCTCAAAGAC
Mouse *TNFα* reverse primer: AGATAGCAAATCGGCTGACG
